# Impact of daily, oral pre-exposure prophylaxis on the risk of bacterial sexually transmitted infections among cisgender women: a systematic review and narrative synthesis

**DOI:** 10.12688/wellcomeopenres.17457.2

**Published:** 2022-08-15

**Authors:** Vasiliki Papageorgiou, Erica Crittendon, Flavien Coukan, Bethan Davies, Helen Ward

**Affiliations:** 1 Patient Experience Research Centre, School of Public Health, Imperial College London, London, W2 1PG, UK; 2 National Institute for Health and Care Research Applied Research Collaboration Northwest London, Chelsea and Westminster Hospital, London, SW10 9NH, UK; 3 Department of Epidemiology and Biostatistics, School of Public Health, Imperial College London, London, W2 1PG, UK; 4 National Institute for Health and Care Research Imperial Biomedical Research Centre, London, W2 1NY, UK; 5 MRC Centre for Global Infectious Disease Analysis and Abdul Latif Jameel Institute for Disease and Emergency Analytics, Imperial College London, London, W2 1PG, UK

**Keywords:** pre-exposure prophylaxis, bacterial sexually transmitted infections, women’s health, health risk behaviors, HIV prevention, systematic review

## Abstract

**Background: **There are concerns that the use of pre-exposure prophylaxis (PrEP) may result in an increased incidence of sexually transmitted infections (STIs). Evidence for this is mixed and has mostly been based on reviews focussed on gay and bisexual men and transgender women, while none have summarised evidence in cisgender women.

**Methods: **We conducted a systematic review to explore whether daily, oral PrEP use is associated with changes in bacterial STI occurrence (diagnoses or self-reported) and/or risk among HIV seronegative cisgender women (ciswomen). The quality of evidence was assessed using the Grading of Recommendations, Assessment, Development and Evaluation (GRADE) tool.

**Results: **We included 11 full text articles in a narrative synthesis, with the studies published between 2012 and 2021. The studies were mostly based in Africa (n=7, 63.6%) and reported on 3168 ciswomen using PrEP aged 16–56 years. Studies had marked differences in variables, including measurements and definitions (e.g., STI type) and limited data available looking specifically at ciswomen, principally in studies with both male and female participants. The limited evidence suggests that PrEP use is not associated with increased STI rates in ciswomen generally; however, adolescent girls and young women in Sub Saharan Africa have a higher prevalence of bacterial STIs prior to PrEP initiation, compared to adult ciswomen and female sex workers.

**Conclusions: **We suggest future PrEP research make efforts to include ciswomen as study participants and report stratified results by gender identity to provide adequate data to inform guidelines for PrEP implementation.

**PROSPERO registration: **CRD42019130438

## Introduction

The use of daily antiretroviral pre-exposure prophylaxis (PrEP) has demonstrated efficacy for the prevention of HIV transmission amongst men who have sex with men (MSM), transgender women and heterosexual couples ^1– 6^. PrEP can be administered as a daily, oral tablet or long-acting injection, with global estimates indicating that approximately 925,000 people were enrolled on PrEP in December 2020, with just under a quarter (22%) based in the United States.

A theoretical ‘syndemic’ relationship has been described between HIV prevention and other sexually transmitted infections (STIs), although the causal connection between the two remains unclear ^7^. There is a perceived risk between PrEP uptake, increased condomless sex and an increased risk of STIs being reported ^8– 10^. Among PrEP users, there have been reports of actual or intended changes in risks after uptake, including increased condomless sex acts and multiple partners ^11, 12^, and increased STI incidence ^13^. For instance, a systematic review and meta-analysis of 17 studies ^14^ describe a 24% increased pooled risk of any STI diagnosis following PrEP use by HIV-negative MSM and transgender women [OR, 1.24; 95% CI 0.99, 1.54; *p* = 0.059]. Ong *et al.* ^15^ undertook a random effects meta-analysis of any bacterial STI and reported an overall pooled incidence of 72.2 per 100 person-years (95% CI 60.5, 86.2) following PrEP initiation.

The findings from reviews are predominantly based on open-label clinical trials, limiting the application of results outside a controlled setting ^16– 18^. It is unclear what may be driving differences in risk seen among PrEP users however some theories have been postulated. Firstly, that participants of PrEP trials may be more likely to engage in ‘risky’ sexual practices and, subsequently, be generally more likely to acquire STIs ^10, 14, 19^. For instance, an intended benefit of PrEP use is the freedom for people to have condomless sex, if they wish, without the risk of acquiring HIV ^20^. Secondly, temporal changes suggest an increased trend in the risk of STIs amongst the general population; some argue that increased STI rates were observed before the introduction of PrEP ^10^. Alternatively, some studies have reported no significant difference in either STI incidence nor risk ^1– 3, 16, 21– 25^. Overall, the evidence for any causal relationship between PrEP use and increased risk remains inconclusive ^10, 15, 26, 27^.

Reported changes in STI incidence following PrEP initiation could also be attributed to the sexual risk context. Factors such as partnership practices (e.g. multiple sexual partners), behaviours (e.g. condom use) ^9^, the socio-structural context (e.g. transactional, ‘survival’ sex or mobility) and gender identities or relationship dynamics ^9, 28– 30^ may, in turn, directly or indirectly influence the likelihood of an individual acquiring a bacterial STI. In addition, the socio-political context and nature of the healthcare system could influence the frequency of screening. Providers’ perceptions of risk profiles of patients or educational initiatives could influence STI testing decisions, introducing a detection bias of predominantly asymptomatic STIs within a particular population ^31^. Thus, the association between PrEP and risk is context-specific, whether by country, setting or study design ^10^; population group ^28– 30^; or the availability of other prevention methods ^16, 17^.

Systematic reviews and meta-analyses exploring the impact of PrEP on STI acquisition have primarily focussed on MSM and often subsume transgender women who have sex with men within analyses, report small sample sizes of ciswomen or group ciswomen with other ‘non-MSM’ populations ^15, 26, 32^. This limits the generalisability and interpretation of findings due to differing psychosocial factors, including behavioural vulnerabilities ^33^. The values, preferences and acceptability of PrEP among women have been previously explored, however few clinical trials have reported results specifically on ciswomen, despite accounting for almost half of new HIV infections in adults globally ^19, 34^. For instance, there is a well-established underrepresentation of pregnant, breastfeeding and women of childbearing potential in PrEP trials who are either explicitly excluded in study protocols or unenrolled if a pregnancy occurs during the study, ultimately limiting the acquisition of safety data ^35^. Ancillary studies of clinical trials with female participants have begun to explore the population sexual risk context and its influence on PrEP adherence ^30, 36– 38^. One study ^39^ reports no evidence of any change in sexual behaviour among cisgender female participants of the Partners Demonstration Project, however this study does not report STI diagnoses.

We aim to synthesise evidence from the published literature on the association between oral pre-exposure prophylaxis and the risk of bacterial sexually transmitted infections among cisgender women (gender identity is aligned with sex assigned at birth), including the impact of PrEP on sexual behaviour.

## Methods

The review is registered on PROSPERO (registration: CRD42019130438, 12 April 2019) and we use Preferred Reporting Items for Systematic Reviews and Meta-Analyses (PRISMA) guidelines to report the findings ^40, 41^. A protocol was developed and followed but not published ^41^.

### Eligibility criteria

We included open-label RCTs, demonstration and implementation projects and observational studies. We assessed each article according to our inclusion criteria as stated in our PICO statement ^41^.

Ciswomen were defined to be over the age of 15 to align with UNAIDS statistics. We excluded studies if they solely related to perceptions, acceptability or willingness to take PrEP, rather than actual PrEP use or if they exclusively measured HIV acquisition during PrEP use, rather than STI, as previously described ^14^. To be eligible for inclusion, studies had to include at least three-months follow-up of STI diagnoses, which could be reported as a composite measure (i.e., including non-bacterial STIs).

We included publications that were full-text, peer-reviewed journal articles, conference abstracts or grey literature published in English, with no restrictions placed on publication date, status, and geographic location.

### Search strategy

Two authors (VP, EC): (1) used the search strategy ^41^ to search MEDLINE and EMBASE *via* Ovid, Cochrane CENTRAL and Web of Science from creation to 08/04/19; (2) manually searched trial databases including the EU Clinical Trials Register; ClinicalTrials.gov; WHO International Clinical Trials Registry Platform (ICTRP); Australian New Zealand Clinical Trials Registry (ANZCTR); and the ISRCTN from creation to 17/04/19; (3) conducted a hand search of conference abstract databases of the Conference on Retroviruses and Opportunistic Infections (CROI); British HIV Association (BHIVA); AIDS Impact; IAS Conference on HIV Pathogenesis, Treatment and Prevention; and International AIDS Conference *via* Abstract Archive, from creation to 25/04/19. Two authors (VP, FC) updated the search on 30/10/20 and hand searches on 03/11/20.

### Study selection

Two authors (VP/EC) independently used a screening tool for the first 30 articles to cross-check for consistency in the process and then independently screened titles and abstracts for eligibility. We imported all references into Covidence (RRID:SCR_016484) and conducted screening of titles, abstracts and full texts. Any reasons for excluding studies were noted and one author (VP) contacted study authors where full-text articles were unavailable. Duplicate records were also excluded during screening.

If both conference abstracts and journal articles were reported for the same study, the most recent publication was included. If the selected study was a clinical trial, the most recent publication of results linked to the register was screened. Any conflicts were discussed and resolved by consensus.

### Data extraction

We used a data extraction template ^41^ in Microsoft Excel (Version 2112) (RRID:SCR_016137) and contacted study authors to provide a breakdown of data by gender, if the results were reported as a combined dataset (e.g., heterosexual couples). The following data were recorded: (1) study design and characteristics; (2) participant demographics and baseline characteristics; (3) key findings and outcome measures; (4) follow-up time; and (5) assessment of study quality.

### Risk of bias

The quality of evidence was assessed using the Grading of Recommendations, Assessment, Development and Evaluation (GRADE) tool ^41, 42^ taking into account study limitations (e.g., risk of bias), imprecision of the effect estimates, inconsistency of results (or heterogeneity), indirectness of evidence and risk of reporting (or publication) bias ^43^. For observational studies (or non-randomised studies of interventions), grading is completed by rating upwards (starting from ‘low quality’) whereas randomised controlled trials have a ‘high-certainty’ rating to begin with ^42^. Assessments range across four levels of quality: high, moderate, low and very low and indicate whether further research would “change our confidence in the estimate of effect” or an association of interest ^42, 43^. 

### Synthesis of results

We planned to report STI incidence rates with 95% confidence interval for each study presented on a forest plot. However, due to methodological heterogeneity between the studies identified, findings are presented as a narrative synthesis.

## Results

**Figure 1.**
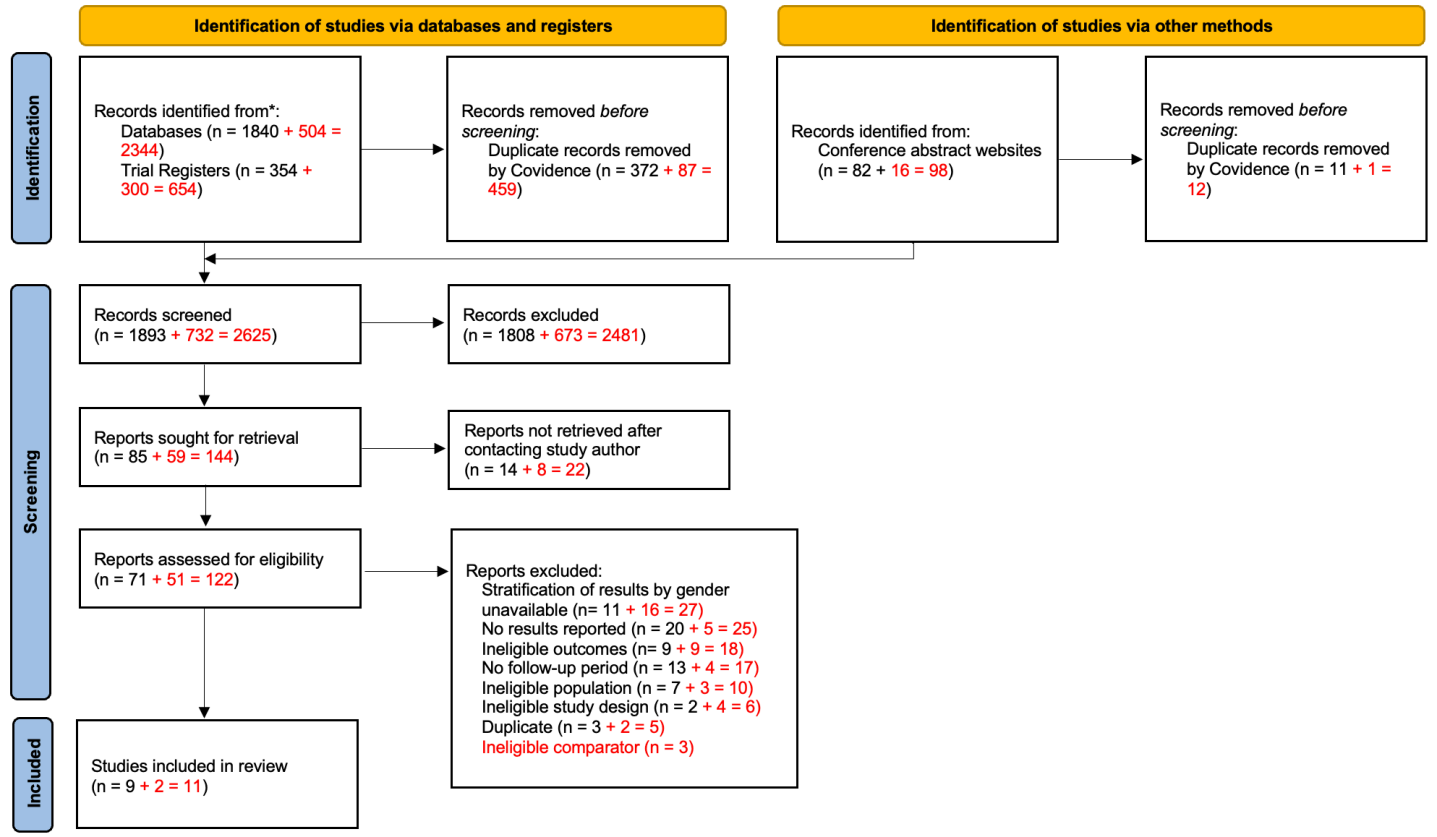
Preferred Reporting Items for Systematic Reviews and Meta-Analyses (PRISMA) 2020 flow diagram. Adapted from Page *et al.* ^40^ We present both search methods together as we did not record these separately when conducting our review in 2019 as it was not a requirement of previous PRISMA guidelines. Text in red indicates updated search (November 2020).

Following the removal of duplicates, we screened a total of 2625 studies. Of these, 122 full-text studies were assessed for eligibility and 11 studies were included in the review ^44– 54^. Detail of our search is outlined in Figure 1.

### Study characteristics

The 11 included studies were published between 2012 and 2021 ( Table 1). Six studies were observational in design ^44– 47, 51, 54^, three were demonstration projects ^48, 49, 53^ and two were RCTs ^50, 52^. Sub-groups of the studies included adolescent girls and young women and female sex workers. Seven of the studies were conducted in Sub Saharan Africa ^44, 46, 47, 49– 52^, three in the United States ^45, 48, 54^, and one in Taiwan ^53^. In total, the studies followed 3168 ciswomen (range 11–1062 participants) enrolled to or using PrEP and aged between 16 to 56 years old, with follow-up for an average of 1.5 years. Loss to follow-up ranged from 14% to 91%.

**Table 1.** Characteristics of included studies in the review.

Study	Project/ Clinic	Design	Location	Participants * (PrEP users/ population screened, %)	Participant age in years [median (IQR)]	Reported outcomes (measurement tool)	Follow-up period	Lost to follow-up	Publication type	Study quality ^§§§^
**Blumenthal ** ***et al*., 2020 ^48^**	PrEP Adherence Enhancement Guided by Individualized Texting and Drug Levels for Women (AEGiS)	Demonstration project	Los Angeles and San Diego (USA)	136/167 cisgender women, 81.4%	Mean 40 (SD=11)	STI infections (lab testing); number of sexual partners (self-report)	4, 12, 24, 36 and 48 weeks	37/136 ^†^, 27.2%	Conference abstract/ poster	⊕ Very low
**Clement ** ***et al*.,** **2021 ^54^^‡^**	Two PrEP clinics (within academic hospital and a community health centre)	Observational (retrospective cohort)	Durham County, North Carolina (USA)	23/271 adults, 8.5%	Mean 33.2 ^§^	STI infection (chart review - clinical/lab testing)	51 months	18/23, 78.3%	Journal article	⊕ Very low
**Delany-** **Moretlwe ** ***et al*., 2019 ^50^**	Evaluation of Daily Oral PrEP as a Primary Prevention Strategy for Young African Women: A Vanguard Study (HPTN 082)	RCT	Cape Town, Johannesburg (South Africa) and Harare (Zimbabwe)	412 AGYW	21	Chlamydia, gonorrhoea, and syphilis diagnoses; (vaginal swabs/urine sample); primary sexual partner (self-report); consistent condom use (self-report)	6 and 12 months	Not reported	Conference abstract/oral presentation	⊕ Very low
**Eakle *et al*., ** **2017 ^44^**	Embedded within Sex Worker Programme (SWP)	Observational (prospective demonstration project)	Johannesburg and Pretoria (South Africa)	219/692 FSWs, 31.6%	28.9 (18.0 to 55.54)	STI infections (screening); number of sexual partners (self-report); consistent condom use (self-report)	1, 3, 6, 9 and 12 months	156/219 **, 71.2%	Journal article	⊕ Very low
**Giguère ** ***et al*., 2019 ^46^**	Prospective early ART (E-ART) and PrEP demonstration project	Observational (prospective demonstration project)	Cotonou (Benin)	255/256 FSWs, 99.6% ^††^	Mean 32.5 (SD=9.2)	STI infections (vaginal swabs); consistent condom use (self-report and vaginal swabs)	6, 12, 18, and 24 months	135/255 ^‡‡^, 52.9%	Journal article	⊕⊕ Low
**Gill *et al*., ** **2019 ^49^**	3Ps for Prevention Study (Perception, Partners, Pills) (3P)	Demonstration project	Cape Town (South Africa)	200 AGYW	19 (17 to 21)	Chlamydia and gonorrhoea diagnoses (screening)	6 months	Not reported	Conference abstract	⊕ Very low
**Maljaars ** ***et al*., 2017 ^47^**	PlusPills	Observational (cohort)	Cape Town and Soweto (South Africa)	98/148 adolescents, 66.2%	18.0 (16.0 to 19.0)	STI infections (screening); consistent condom use (self-report)	4, 8 and 12 weeks	40/98, 40.8% ^§§^	Journal article	⊕ Very low
**Stewart ** ***et al*., 2019 ^51^**	Prevention Options for Women Evaluation Research (POWER) Study	Observational (prospective cohort)	Kisumu (Kenya)	708 AGYW	Not reported	Chlamydia and gonorrhoea diagnoses (urine sample)	Up to 36 months (quarterly)	643/708, 90.8%	Conference abstract	⊕ Very low
**Tabidze ** ***et al*., 2018 ^45^**	Howard Brown Health Centre	Observational (retrospective cohort)	Chicago (USA)	44/2984 adults, 1.5%	32 ^§^	Chlamydia, gonorrhoea, and syphilis diagnoses; (testing) number of sexual partners (self-report); consistent condom use (self-report)	28 months	Not reported	Conference abstract /poster	⊕ Very low
**Van Damme ** ***et al*., 2012 ^52^**	FEM-PrEP	RCT	Bondo (Kenya); Bloemfontein and Pretoria (South Africa); Arusha (Tanzania)	1062/4163 *** women, 25.5%	23 (18 to 35)	Chlamydia, gonorrhoea, and syphilis diagnoses (cervical swabs and blood test); number of sexual partners (self-report); consistent condom use (self-report)	Up to 60 weeks (4-week intervals)	148/1062 ^†††^, 13.9%	Journal article	⊕⊕ Low
**Wu *et al*., ** **2018 ^53^**	Taiwan Demonstration Project	Demonstration project	Taipei, Taoyuan City, Tainan City and Kaohsiung City (Taiwan)	11/302 adults, 3.6% ^‡‡‡^	34.6 (31.7 to 43.6)	Syphilis diagnoses (lab tests); number of sexual partners (self-report); consistent condom use (self-report)	12 months (quarterly)	Not reported	Conference abstract/ poster	⊕ Very low

Abbreviations: AGYW: adolescent girls and young women; FSWs, female sex workers; RCT, randomised controlled trial ^*^ Defined as cisgender women enrolled to, or using, daily PrEP. Total population screened may include male participants or HIV positive (subsequently not enrolled). ^†^ Reported 52 early terminations by week 48; 37 of which were lost to follow-up, 15 were formal withdrawals (with reasons listed). ^‡^ During updated search, paper was published ahead of print; therefore, year of publication is after date of search ^§^ Includes all study participants (i.e. not only cisgender women). ^**^ 156 FSWs were lost to follow up / had no exit visit. Study authors also reported 4 FSWs who withdrew from study. ^††^ One person excluded from analysis due to missing data. ^‡‡^ Study authors also report attrition regarding number of participants not followed due to administrative censorship (n=90) and withdrawals (n=135) by month 24. ^§§^ Calculated by VP ^***^ 4163 women screened; 2120 women underwent randomisation, of which 1058 were assigned placebo and 1062 PrEP. ^†††^ Study authors also report PrEP users who discontinued early (n=59) ^‡‡‡^ 3 women were taking daily PrEP, 8 were taking on-demand and mixed PrEP ^§§§^Study quality ranked as very low (⊕), low (⊕ ⊕), moderate (⊕⊕⊕) and very high (⊕⊕⊕⊕) quality.

### Synthesis of results: sexually transmitted infections

Table 2 provides a summary of baseline prevalence of STIs and reported risk after PrEP initiation. The definition of STIs across studies varied; some encompassed broader terminology of ‘STI infections’ and included viral types such as genital warts ^44^, while others used site-specific types such as genital gonorrhoea ^45^.

**Table 2.** Reported risk of bacterial STIs, among ciswomen PrEP users, at baseline and following PrEP initiation within included studies. Abbreviations: STI, sexually transmitted infection.

Study	STI diagnoses at baseline	STI diagnoses at follow-up	% change *	Rate (per 100 person years)
**Blumenthal *et al*., 2020 ^48^**	STI infection (12/136, 8.8%)	Bacterial STIs (n=4)	-	5/100py (95% CI 2, 10)
**Clement *et al*., 2021 ^54^**	-	1 or more new STIs (2/23, 8.7%)	-	
**Delany-Moretlwe *et al*., ** **2019 ^50^**	Chlamydia (120/412, 29%) *	119/412, 28.9% (n=79: new infections)	-	29.6/100py (95% CI 24.3, 35.4)
	Gonorrhoea (33/412, 8%) *	48/412, 42.3% (n= 41: new infections)	+428.8%	11.8/100py (95% CI 8.7, 15.7)
	Reactive syphilis serology (8/412, 2%) *	-	-	-
**Eakle *et al*., 2017 ^44^**	STI infections ^†^ (1/17, 5.9%)	**3 months** (0/3, 0%)	-100%	-
		**6 months** (0/3, 0%)	-100%	-
		**9 months** (0/2, 0%)	-100%	-
		**12 months** (0/5, 0%)	-100%	-
		**15 months** (0/0, 0%)	-100%	-
		**18** **months** (0/0, 0%)	-100%	-
		**21 months** (0/0, 0%)	-100%	-
**Giguère *et al*., 2019 ^46^**	STI infections ^‡^ (39/249, 15.7%; 95% CI 11.8, 21.0)	**6 months** (8.4%; 95% CI 4.5, 15.5)	-46.5%	-
		**12 months** (13.2%; 95% CI 7.1, 24.4)	-15.9%	-
		**18 months** (8.6%; 95% CI 2.6, 28.0)	-45.2%	-
		**24 months** (2.1%; 95% CI 0.4, 10.2)	-86.6%	-
**Gill *et al*., 2019 ^49^**	Curable STI infection ^§^ (66/200, 33%)	-	-	52/100py
	Chlamydia (50/200, 25%) *	**6 months** 24/39, 62% new infections *	+148%	42/100py
	Gonorrhoea (22/200, 11%) *	**6 months** 10/13, 77% new infections *	+600%	14/100py
**Maljaars *et al*., 2017 ^47^**	STI infections ** (27/58, 46.6%)	**12 weeks** (19/54, 35.2%)	-24.5%	-
**Stewart *et al*., 2019 ^51^**	Chlamydia (120/708, 17%) *	-	-	40/100py
	Gonorrhoea (56/708, 8%) *	-	-	12.3/100py
**Tabidze *et al*., 2018 ^45^^††^**	All types of syphilis ^‡‡^ (0%)	**1 year** (0%)	-	-
	Genital gonorrhoea (0%)	**1 year** (3.3%)	-	-
	Rectal gonorrhoea (9.1%)	**1 year** (0%)	-100%	-
	Pharyngeal gonorrhoea (4%)	**1 year** (3.6%)	-3.8%	-
	Genital chlamydia (13.5%)	**1 year** (6.7%)	-50.6%	-
	Rectal chlamydia (0%)	**1 year** (0%)	-	-
	Pharyngeal chlamydia (0%)	**1 year** (0%)	-	-
**Van Damme *et al*., 2012 ^52^**	Gonorrhoea (56/939, 6.0%)	**Week 60** (4.9%)	-18.3%	-
	Chlamydial infection (142/939, 15.1%)	**Week 60** (13.3%)	-11.9%	-
	Syphilis (21/1060, 2.0%)	-	-	-
**Wu *et al*., 2018 ^53^^§§^**	Syphilis (0/11, 0%)	Syphilis (1/11, 9.1%)	-	-

^*^ Numerator backcalculated by VP ^†^ STIs recorded: genital ulcer disease, genital warts, herpes, vaginal candidiasis, vaginal discharge, abscess, pelvic inflammatory disease (PID). STI infection recorded = PID/Total STIs. Presented in original maunscript’s supplementary file (see Table H). ^‡^ Positive tests for trichomoniasis (1/250, 0.4%), chlamydia (14/249, 5.6%), gonorrhoea (28/249, 11.2%) ^§^ Includes chlamydia, gonorrhoea and trichomoniasis. ^**^ STIs tested: Herpes Simplex Virus-2, *Chlamydia trachomatis* and *Nesisseria gonorrhoeae* ^††^ Study authors contacted for further data as this was provided in a conference abstract/poster. Outcome is changes between year before and after PrEP start. ^‡‡^ Syphilis defined as primary and secondary, early latent and late latent. ^§§^ Study authors contacted for further data as this was provided in a conference abstract / poster. Data breakdown not provided by type of PrEP (n=3, daily; n=8, on-demand and mixed). Wu *et al.* includes all women on the study (n=11) and therefore includes on-demand dosage. *** Percentage change calculation = [(follow-up % - baseline %) / baseline %] * 100

***Baseline prevalence*.** The baseline prevalence of STI infections in five studies, using a broader definition, ranged between 6% to 47% ^44, 46– 49^. Baseline prevalence of chlamydia was reported in six studies and ranged from 6% to 29% ^45, 46, 49– 52^. Two studies with a population of adolescent girls and young women reported a baseline prevalence of 25% and 29% respectively ^49, 50^. Gonorrhoea diagnoses at baseline were reported across six studies and ranged from 6% to 11% ^45, 46, 49– 52^. Syphilis diagnoses, reported in four studies, were low at baseline (prevalence ranging from 0% to 2%) ^45, 50, 52, 53^.

***Follow-up prevalence*.** Overall, the prevalence of diagnosed STIs reduced or remained stable during follow-up. However, there were no significant trends nor consistent methods for the measurement and reporting of STIs. Giguère *et al*. ^46^ found that STI prevalence (defined as trichomoniasis, chlamydia and gonorrhoea) declined from 15.7% (95% CI 11.8, 21.1) at baseline to 2.1% (95% CI 0.4, 10.2) at 24-months, but found no difference between baseline and 12 months, nor 12 to 24 months. Eakle *et al*. ^44^ reported bacterial STI diagnoses (including pelvic inflammatory disease, which can be used as an indicator of untreated gonorrhoea and chlamydia), at three-month intervals (up to 21 months) and also found no significant difference in diagnoses at baseline and follow-up.

Gonorrhoea diagnoses decreased during follow-up in most studies; for instance, Maljaars *et al*. ^47^ reported a 24.5% reduction in STI infections (including gonorrhoea) 12-weeks following PrEP initiation. Van Damme *et al*. ^52^ found gonorrhoea infections declined from 6.0% at baseline to 4.9% at follow-up among PrEP users (5.5% to 3.2% in placebo arm, *P=*0.25) and chlamydial infections from 15.1% to 13.3% (12.9% to 12.0% in placebo arm, *P=*0.65). However, 148 women (13.9%) were lost to follow-up ^52^. One study ^45^ reported a small increase of genital gonorrhoea at one-year (0% to 3.3%) but this was based on a small sample (n=44), i.e., only 1.5% of the whole study were ciswomen).

***Incident infections during follow-up*.** Three studies calculated the incidence rate of chlamydia diagnoses at between 30 and 42 cases per 100 person-years ^49– 51^. In two studies of adolescent girls and young women, around two-thirds of chlamydia infections were incident, i.e., not present when PrEP started ^49, 50^. Furthermore, three studies that calculated the overall incidence rate of gonorrhoea infections reported these at 12–14 cases per 100 person-years ^49– 51^. In two studies of adolescent girls and young women, over three-quarters of gonorrhoea infections were incident ^49, 50^. Two studies reported subsequent syphilis diagnoses at follow-up; no syphilis was detected during follow-up by Tabidze *et al.* ^45^ and Wu *et al*. ^53^ reported one new case of syphilis among 11 ciswomen using PrEP (including on-demand or mixed dosage).

### Synthesis of results: sexual behaviour

Analysis of reported behaviour showed inconsistent results, with no clear signal of increase in risk following PrEP initiation. Table 3 illustrates risks reported across the studies. Sexual partners were variably defined across the studies; some studies specified by the ‘type’ of sexual partner (e.g., casual, regular, main), with sex workers definitions of ‘client’ also specified (e.g., occasional, regular). Condom use also varied in definition across the studies in terms of ‘consistency’ or ‘condomless sex acts.’ All studies were based on self-reports; however, one study also explored the accuracy (underreporting) of reporting condomless sex by comparing self-reports against a biological assessment (vaginal swabs) which detected traces of prostate-specific antigen (PSA) and Y-chromosomal DNA (Yc-DNA) as biomarkers of recent semen exposure ^46^.

**Table 3.** Self-reported risk (including sexual partners, consistent condom use), among PrEP users, at baseline and following PrEP initiation within included studies *.

Study	Number of sexual partners at baseline [mean (SD)]	Number of sexual partners at follow-up [mean (SD)]	Consistent condom use at baseline	Consistent condom use at follow-up
**Blumenthal *et al*., ** **2020 ^48^**	Number of sex partners in past 3 months [Median (IQR)]: 1 (1-3)	-	-	-
**Delany-Moretlwe ** ***et al*., 2019 ^50^**	Primary sex partner (346/412, 84%) ^†^	-	Never or rarely use condoms (144/412, 35%) ^†^	-
**Eakle *et al*., 2017 ^44^**	Casual partner in past 7-days [0.7 (1.1)]	**3 months** [3 (3.2)]	Casual partner in past 7-days (14/22, 64%)	**3 months** (5/6, 83%)
		**6 months** [1 (1.5)]		**6 months** (4/7, 57%)
		**9 months** [1.4 (1.1)]		**9 months** (5/5, 100%)
		**12 months** [1.5 (1.3)]		**12 months** (3/4, 75%)
	Occasional client in past 7-days [17.5 (21.8)]	**3 months** [13.9 (13.2)]	Occasional client in past 7-days (181/181, 100%)	**3 months** (78/78, 100%)
		**6 months** [23.2 (32.4)]		**6 months** (54/54, 100%)
		**9 months** [24.7 (31.9)]		**9 months** (38/40, 95%)
		**12 months** [25 (22.1)]		**12 months** (33/33, 100%)
	Regular client in past 7-days [22.9 (21.1)]	**3 months** [12.9 (10.3)]	Regular client in past 7-days (179/180, 99%)	**3 months** (80/82, 98%)
		**6 months** [8.3 (7.9)]		**6 months** (58/59, 98%)
		**9 months** [9.1 (8.9)]		**9 months** (49/50, 98%)
		**12 months** [10.7 (9.4)]		**12 months** (31/31, 100%)
	-	-	Main partner in past 7-days (47/144, 33%)	**3 months** (25/62, 40%)
				**6 months** (13/41, 32%)
				**9 months** (14/34, 41%)
				**12 months** (7/27, 26%)
**Giguère *et al*.,** **2019 ^46^**	-	-	Weighted prevalence of unprotected sex in last 2 days (69/254, 27.2%; 95% CI 22.3, 33.2)	**6 months** (18.4%; 95% CI 12.9, 26.3)
				**12 months** (18.1%, 95% CI 11.8, 27.6; *P*=0.04)
				**18 months** (30.3%; 95% CI 15.5, 59.1)
				**24 months** (34.2%; 16.6, 70.5; *P=*0.42)
			Weighted prevalence of unprotected sex in last 14 days (53.6%; 95% CI 47.7, 60.1)	**6 months** (45.8%; 95% CI 38.3, 54.8)
				**12 months** (48.6%; 95% CI 39.8, 59.5, *P=*0.36)
				**18 months** (49.0%; 95% CI 34.3, 69.9)
				**24 months** (38.7%; 95% CI 18.4, 81.4; *P=*0.49)
**Maljaars *et al*., ** **2017 ^47^**	-	-	Inconsistent condom use (44/58, 75.9%)	Data unavailable - not stratified by gender
			Condom use during last sexual act (39/58, 67.2%)	Data unavailable - not stratified by gender
**Tabidze *et al*., ** **2018 ^45^** ^‡^	Median = 2	**12 months** (median=6, *P=*0.18)	Never/sometimes using condoms (77.78%)	**12 months** (75%, *P=*0.32, effect sample size = 8)
**Van Damme *et al*., ** **2012 ^52^**	Partners in past week (mean = 1.0, median = 1, range = 0-6)	-	Sex without condom in past week (mean = 1.9, median = 1, range = 0-25)	-
**Wu *et al*., 2018 ^53^** ^§^	0 regular sexual partners (1/11, 9.1%)	-	Consistent condom use in past 3-months (6/11, 54.5%)	-
	0–3 regular sexual partners (9/11, 81.8%)			
	≥4 regular sexual partners (1/11, 9.1%)			
	0 casual sexual partners (6/11, 54.5%)			
	0–3 casual sexual partners (3/11, 27.3%)			
	≥4 casual sexual partners (2/11, 18.2%)			

^*^ Some included studies did not provide follow-up data but are presented here to provide context when interpreting findings ^†^ Numerator backcalculated by VP ^‡^ Study authors contacted for further data as this was provided in a conference abstract / poster. Outcome is changes between year before and after PrEP start. ^§^ Study authors contacted for further data as this was provided in a conference abstract / poster. Data breakdown not provided by type of PrEP (n=3, daily; n=8, on-demand and mixed).

At baseline, the average number of sexual partners differed between studies (range: 0.7, 22.9). Consistent condom use at baseline ranged from 33% to 100%. Only three studies ^44– 46^ provided data on sexual behaviour at both baseline and follow-up. Eakle *et al*. ^44^ describe an increase in the mean number of casual sexual partners [0.7 (SD=1.1) to 1.5 (SD=1.3)] and occasional clients [17.5 (SD=21.8) to 25.0 (SD=22.1)] in the past seven-days, when comparing baseline to 12-months after PrEP initiation. They report a decrease in the mean number of regular clients [22.9 (SD=21.1) to 10.7 (SD=9.4)] in the past seven-days, when comparing baseline to 12-months after PrEP initiation ^44^. Tabidze *et al*. ^45^ describe a non-significant increase in median number of sexual partners from two to six following 12-months PrEP use ( *P=*0.18).

Generally, consistent condom use was unchanged or improved. One study ^46^ reported a significant reduction in unprotected sex after 12 months, compared to baseline (27.2% to 18.1%, *P=*0.04). Two studies ^45, 47^ found no change and Eakle *et al*. ^44^ had insufficient data due to loss to follow-up.

### Quality assessment

Overall, the quality of evidence was very low ^41^. Nine studies (81.8%) scored very low and two studies (18.2%) low quality when using the GRADE assessment. Firstly, the sample size of ciswomen using daily PrEP in four of the studies ^45, 47, 53, 54^ was small (range: 3–98) which will have resulted in imprecise measurements. Secondly, high loss to follow-up (ranging from 13.9% to 90.8%) of participants was reported in most studies (n=6), introducing substantial risk of bias. Thirdly, the presentation of data limited the ability to determine any significant changes to risk. For instance, Maljaars *et al*. ^47^ did not stratify behaviour outcomes by gender, therefore it is not possible to determine whether any changes in condom use are influenced by gender.

## Discussion

We found no consistent evidence that PrEP use increased the risk of ciswomen acquiring bacterial STIs, with some studies indicating that it may be associated with reduced risk. This aligns with similar findings in MSM and transgender women, described in observational studies ^16, 24^, demonstration projects ^21, 22, 25^, pilot studies ^23^ and RCTs ^1– 3^. Additionally, our review found no clear evidence that PrEP use results in an increased likelihood of engaging in an action that can make ciswomen more vulnerable to acquiring an STI. We found that adolescent girls and young women in sub Saharan Africa have a high prevalence and incidence of bacterial STIs (particularly chlamydia and gonorrhoea) which is linked to higher vulnerabilities based on age-disparate sex, transactional sex, gender norms and lifetime gender-based violence ^55– 58^.

Increasingly diverse methods of PrEP (including delivery and dosage) provide an increasingly personalised approach to HIV prevention ^7^. Our review focussed on oral PrEP however, other methods for HIV prevention include the vaginal ring (either dapivirine or tenofovir), which has been found to be an acceptable method among women, with high continuation rates seen in both observational studies and randomised controlled trials (RCTs) ^59– [Bibr ref-62]^ as well as injectable PrEP ^63^. The HPTN 084 study found 8-weekly injections of long-acting PrEP (cabotegravir) to be 89% more effective (HR 0.11; 95% CI 0.01, 0.31) among ciswomen in sub-Saharan Africa than the daily, oral PrEP (tenofovir/emtricitabine) ^63^.

### Strengths and limitations

To our knowledge, this is the first exploration of this research question in this population group. While the included studies had widely varying methods, they all followed participants for at least three months. We used a systematic approach to critically summarise and review the limited literature that currently exists on this topic. There are several limitations; firstly, despite an extensive literature search, we found few studies examining STI risk following PrEP initiation which contrasts with the evidence on PrEP efficacy. Our review did not include studies using other modalities of PrEP (e.g., injectable, vaginal ring); therefore, we are unable to draw conclusions on whether different modalities effect STI risk or changes in or changes in sexual behaviour and would need separate studies to review these relationships. Secondly, several of the included studies were very small and all were assessed as ‘low’ or ‘very low’ quality. We also did not search specific clinical trials registers for trials conducted in Africa (e.g., the South African Clinical Trials Register (SANCTR) or Pan African Clinical Trials Registry (PACTR)) which may have introduced a publication bias relating to population setting. Thirdly, there is marked variability in the measurement of STI outcomes (specifically timing, types, and composite endpoints) and sexual behaviour risk, which are essential to interpret patterns and causal pathways of STI acquisition to guide public health interventions. For instance, STI diagnoses varied across studies and are likely to have been influenced by attrition bias; notably in the FEM-PrEP study ^52^, where less than half of participants had a pelvic examination which would then confirm the self-reported data on risk. Finally, there were high numbers of loss to follow-up (range: 14%–91%) across studies; for instance, Wu *et al*. ^53^ explained that loss to follow-up was influenced by women who could not afford or were unwilling to pay for PrEP as participants were only offered a maximum of 105 pills for a year.

Due to the heterogeneity in study designs and outcome measures, it was not appropriate to conduct a meta-analysis, restricting our synthesis to a narrative review. Just over one-quarter (n=3/11, 27.3%) of the included studies reported sexual behaviour at follow-up and baseline, therefore we were unable to measure the full extent of this potential impact.

### Study implications

Our findings suggest that, like previous reviews including MSM and transgender women, there is no evidence that ciswomen using PrEP have a changed risk of bacterial STIs; however, adolescent girls and young women in sub–Saharan Africa had very high prevalence of bacterial STIs at PrEP initiation. Overall, we found very limited and low-quality evidence, making it difficult to draw any solid conclusions. This highlights an important issue of gender data biases in clinical research design, conduct and reporting, and specifically HIV prevention trials ^64– 66^. Despite this, the provision of PrEP presents an opportunity to engage women in programmes to prevent and treat STIs, providing opportunities for sexual health promotion and advice ^15, 67, 68^. This is particularly important for those populations of adolescent girls and young women in sub-Saharan Africa with a high burden of bacterial STIs ^69, 70^.

## Conclusions

Based on this review, there is insufficient evidence to show whether PrEP use is associated with increased STI diagnoses for ciswomen. Specifically, the quality of evidence from included studies were limited and emphasises a need for larger scale studies of cisgender women in different settings which also measure sexual behaviour including condom use and number of sexual partners at both baseline and during follow-up. We emphasise the need for larger PrEP studies with ciswomen using standard periods of follow-up that align with testing guidelines. We also suggest consistent definitions of STIs, stratification of data by gender identity and validated and standardised methods for measuring risk are used in these studies to provide more robust data to help inform PrEP implementation guidelines.

## Data availability

### Extended data

Zenodo: Extended data for ‘Impact of daily, oral pre-exposure prophylaxis on the risk of bacterial sexually transmitted infections among cisgender women: a systematic review and narrative synthesis’. https://doi.org/10.5281/zenodo.5827582 ^41^

This project contains the following extended data:

•   Additional file 1: PRISMA 2020 checklist.docx

•   Additional file 2: Systematic review protocol.pdf

•   Additional file 3: PICO criteria.docx

•   Additional file 4: Search strategy.pdf

•   Additional file 5: Standardised data extraction template.xlsx

•   Additional file 6: GRADE assessment of included studies.docx

### Reporting guidelines

Zenodo: PRISMA checklist for ‘Impact of daily, oral pre-exposure prophylaxis on the risk of bacterial sexually transmitted infections among cisgender women: a systematic review and narrative synthesis’. https://doi.org/10.5281/zenodo.5827582 ^41^

Data are available under the terms of the Creative Commons Attribution 4.0 International license (CC-BY 4.0).

## References

[1] Grant RM Lama JR Anderson PL : Preexposure chemoprophylaxis for HIV prevention in men who have sex with men. *N Engl J Med.* 2010;363(27):2587–99. 10.1056/NEJMoa1011205 21091279 PMC3079639

[2] McCormack S Dunn DT Desai M : Pre-exposure prophylaxis to prevent the acquisition of HIV-1 infection (PROUD): effectiveness results from the pilot phase of a pragmatic open-label randomised trial. *Lancet.* 2016;387(10013):53–60. 10.1016/S0140-6736(15)00056-2 26364263 PMC4700047

[3] Molina JM Capitant C Spire B : On-Demand Preexposure Prophylaxis in Men at High Risk for HIV-1 Infection. *N Engl J Med.* 2015;373(23):2237–46. 10.1056/NEJMoa1506273 26624850

[4] Baeten JM Donnell D Ndase P : Antiretroviral prophylaxis for HIV prevention in heterosexual men and women. *N Engl J Med.* 2012;367(5):399–410. 10.1056/NEJMoa1108524 22784037 PMC3770474

[5] National Institute for Health and Care Excellence: Pre-exposure prophylaxis of HIV in adults at high risk: Truvada (emtricitabine/tenofovir disoproxil) (ESNM78).2016. Reference Source

[6] Thigpen MC Kebaabetswe PM Paxton LA : Antiretroviral preexposure prophylaxis for heterosexual HIV transmission in Botswana. *N Engl J Med.* 2012;367(5):423–34. 10.1056/NEJMoa1110711 22784038

[7] Stewart J Baeten JM : HIV pre-exposure prophylaxis and sexually transmitted infections: intersection and opportunity. *Nat Rev Urol.* 2022;19(1):7–15. 10.1038/s41585-021-00527-4 34697493 PMC9249100

[8] Gandhi M Spinelli MA Mayer KH : Addressing the Sexually Transmitted Infection and HIV Syndemic. *JAMA.* 2019;321(14):1356–8. 10.1001/jama.2019.2945 30964514 PMC6615736

[9] Golub SA Pena S Fikslin RA : Partners, not condom use, drive STI rates among PrEP users in community health center. *Topics in Antiviral Medicine.* 2018;26(Supplement 1):468s.

[10] Montano MA Dombrowski JC Barbee LA : Changes in sexual behavior and STI diagnoses among MSM using PrEP in Seattle, WA.Conference on Retroviruses and Opportunistic Infections (CROI); Seattle, Washington,2017.

[11] Lal L Audsley J Murphy DA : Medication adherence, condom use and sexually transmitted infections in Australian preexposure prophylaxis users. *AIDS.* 2017;31(12):1709–14. 10.1097/QAD.0000000000001519 28700394

[12] Marcus JL Hurley LB Hare CB : Preexposure prophylaxis for HIV prevention in a large integrated health care system: adherence, renal safety, and discontinuation. *J Acquir Immune Defic Syndr.* 2016;73(5):540–6. 10.1097/QAI.0000000000001129 27851714 PMC5424697

[13] Hightow-Weidman LB Magnus M Beauchamp G : Incidence and Correlates of Sexually Transmitted Infections Among Black Men Who Have Sex With Men Participating in the HIV Prevention Trials Network 073 Preexposure Prophylaxis Study. *Clin Infect Dis.* 2019;69(9):1597–604. 10.1093/cid/ciy1141 30615169 PMC6792108

[14] Traeger MW Schroeder SE Wright EJ : Effects of Pre-exposure Prophylaxis for the Prevention of Human Immunodeficiency Virus Infection on Sexual Risk Behavior in Men Who Have Sex With Men: A Systematic Review and Meta-analysis. *Clin Infect Dis.* 2018;67(5):676–86. 10.1093/cid/ciy182 29509889

[15] Ong JJ Baggaley RC Wi TE : Global Epidemiologic Characteristics of Sexually Transmitted Infections Among Individuals Using Preexposure Prophylaxis for the Prevention of HIV Infection: A Systematic Review and Meta-analysis. *JAMA Netw Open.* 2019;2(12):e1917134. 10.1001/jamanetworkopen.2019.17134 31825501 PMC6991203

[16] Parsons J Rendina HJ Whitfield T : Changes in rectal STI incidence and behavioral HIV risk before, during, and after PrEP in a national sample of gay and bisexual men in the United States. *J Int AIDS Soc.* 2018;21(S6):e25148.30051631

[17] Traeger MW Asselin J Price B : Changes, Patterns and Predictors of Sexually Transmitted Infections in Gay and Bisexual Men Using PrEP: Interim Analysis from the PrEPX Study. *J Int AIDS Soc.* 2018;21(S6):e25148.30051631

[18] Traeger MW Cornelisse VJ Asselin J : Association of HIV Preexposure Prophylaxis With Incidence of Sexually Transmitted Infections Among Individuals at High Risk of HIV Infection. *JAMA.* 2019;321(14):1380–90. 10.1001/jama.2019.2947 30964528 PMC6459111

[19] Grant RM Anderson PL McMahan V : Uptake of pre-exposure prophylaxis, sexual practices, and HIV incidence in men and transgender women who have sex with men: A cohort study. *Lancet Infect Dis.* 2014;14(9):820–9. 10.1016/S1473-3099(14)70847-3 25065857 PMC6107918

[20] Ward H : S03.1 Impact of HIV PrEP on risk compensation and STI epidemiology – what does the evidence show? *Sexually Transmitted Infections.* 2019;95(Suppl 1):A9–10. 10.1136/sextrans-2019-sti.24

[21] Grinsztejn B Hoagland B Moreira RI : Retention, engagement, and adherence to pre-exposure prophylaxis for men who have sex with men and transgender women in PrEP Brasil: 48 week results of a demonstration study. *Lancet HIV.* 2018;5(3):e136–45. 10.1016/S2352-3018(18)30008-0 29467098

[22] Hosek SG Landovitz RJ Kapogiannis B : Safety and Feasibility of Antiretroviral Preexposure Prophylaxis for Adolescent Men Who Have Sex With Men Aged 15 to 17 Years in the United States. *JAMA Pediatr.* 2017;171(11):1063–71. 10.1001/jamapediatrics.2017.2007 28873128 PMC5710370

[23] Hosek SG Siberry G Bell M : The acceptability and feasibility of an HIV preexposure prophylaxis (PrEP) trial with young men who have sex with men. *J Acquir Immune Defic Syndr.* 2013;62(4):447–56. 10.1097/QAI.0b013e3182801081 24135734 PMC3656981

[24] Lalley-Chareczko L Clark D Conyngham C : Delivery of TDF/FTC for Pre-exposure Prophylaxis to Prevent HIV-1 Acquisition in Young Adult Men Who Have Sex With Men and Transgender Women of Color Using a Urine Adherence Assay. *J Acquir Immune Defic Syndr.* 2018;79(2):173–8. 10.1097/QAI.0000000000001772 29905593

[25] Liu AY Cohen SE Vittinghoff E : Preexposure Prophylaxis for HIV Infection Integrated With Municipal- and Community-Based Sexual Health Services. *JAMA Intern Med.* 2016;176(1):75–84. 10.1001/jamainternmed.2015.4683 26571482 PMC5042323

[26] Freeborn K Portillo CJ : Does pre-exposure prophylaxis for HIV prevention in men who have sex with men change risk behaviour? A systematic review. *J Clin Nurs.* 2018;27(17–18):3254–65. 10.1111/jocn.13990 28771856 PMC5797507

[27] Jenness S Weiss K Goodreau SM : STI incidence among MSM following HIV preexposure prophylaxis: a modeling study.Conference on Retroviruses and Opportunistic Infections (CROI); Seattle, Washington,2017. Reference Source

[28] Corneli A Namey E Ahmed K : Motivations for Reducing Other HIV Risk-Reduction Practices if Taking Pre-Exposure Prophylaxis: Findings from a Qualitative Study Among Women in Kenya and South Africa. *AIDS Patient Care STDS.* 2015;29(9):503–9. 10.1089/apc.2015.0038 26196411 PMC4553377

[29] Davey C Cowan F Hargreaves J : The effect of mobility on HIV-related healthcare access and use for female sex workers: A systematic review. *Soc Sci Med.* 2018;211:261–73. 10.1016/j.socscimed.2018.06.017 29966821

[30] Headley J Lemons A Corneli A : The sexual risk context among the FEM-PrEP study population in Bondo, Kenya and Pretoria, South Africa. *PLoS One.* 2014;9(9):e106410. 10.1371/journal.pone.0106410 25229403 PMC4167553

[31] Spinelli MA Scott HM Vittinghoff E : Provider adherence to pre-exposure prophylaxis monitoring guidelines in a large primary care network. *Open Forum Infect Dis.* 2018;5(6):ofy099. 10.1093/ofid/ofy099 29977959 PMC6016415

[32] Werner RN Gaskins M Nast A : Incidence of sexually transmitted infections in men who have sex with men and who are at substantial risk of HIV infection - A meta-analysis of data from trials and observational studies of HIV pre-exposure prophylaxis. *PLoS One.* 2018;13(12):e0208107. 10.1371/journal.pone.0208107 30507962 PMC6277101

[33] Poteat T German D Flynn C : The conflation of gender and sex: Gaps and opportunities in HIV data among transgender women and MSM. *Glob Public Health.* 2016;11(7–8):835–48. 10.1080/17441692.2015.1134615 26785751 PMC4957661

[34] Koechlin FM Fonner VA Dalglish SL : Values and Preferences on the Use of Oral Pre-exposure Prophylaxis (PrEP) for HIV Prevention Among Multiple Populations: A Systematic Review of the Literature. *AIDS Behav.* 2017;21(5):1325–35. 10.1007/s10461-016-1627-z 27900502 PMC5378753

[35] Joseph Davey DL Bekker LG Bukusi EA : Where are the pregnant and breastfeeding women in new pre-exposure prophylaxis trials? The imperative to overcome the evidence gap. *Lancet HIV.* 2022;9(3):e214–e222. 10.1016/S2352-3018(21)00280-0 35090604 PMC9178651

[36] Kintu A Hankinson SE Balasubramanian R : Sexual Relationships Outside Primary Partnerships and Abstinence Are Associated With Lower Adherence and Adherence Gaps: data From the Partners PrEP Ancillary Adherence Study. *J Acquir Immune Defic Syndr.* 2015;69(1):36–43. 10.1097/QAI.0000000000000538 25942457 PMC4422183

[37] Matthews LT Heffron R Mugo NR : High medication adherence during periconception periods among HIV-1-uninfected women participating in a clinical trial of antiretroviral pre-exposure prophylaxis. *J Acquir Immune Defic Syndr.* 2014;67(1):91–97. 10.1097/QAI.0000000000000246 25118795 PMC4149628

[38] Mujugira A Baeten JM Donnell D : Characteristics of HIV-1 serodiscordant couples enrolled in a clinical trial of antiretroviral pre-exposure prophylaxis for HIV-1 prevention. *PLoS One.* 2011;6(10):e25828. 10.1371/journal.pone.0025828 21998703 PMC3187805

[39] Ortblad KF Stalter R Bukusi EA : No evidence of sexual risk compensation among HIV serodiscordant couples on PrEP. Conference on Retroviruses and Opportunistic Infections (CROI); Seattle, Washington.2019.

[40] Page MJ McKenzie JE Bossuyt PM : The PRISMA 2020 statement: an updated guideline for reporting systematic reviews. *BMJ.* 2021;372:n71. 10.1136/bmj.n71 33782057 PMC8005924

[41] Papageorgiou V Crittendon E Coukan F : Extended Data - Impact of daily, oral pre-exposure prophylaxis on the risk of bacterial sexually transmitted infections among cisgender women: a systematic review and narrative synthesis.2022. 10.12688/wellcomeopenres.17457.1 PMC9391742 36051893

[42] Schünemann HJ Higgins JP Vist GE : Chapter 14: Completing ‘Summary of findings’ tables and grading the certainty of the evidence.In: *Cochrane Handbook for Systematic Reviews of Interventions.* Version 6.3.2022. 10.1002/9781119536604.ch14

[43] Guyatt GH Oxman AD Vist GE : GRADE: an emerging consensus on rating quality of evidence and strength of recommendations. *BMJ.* 2008;336(7650):924–6. 10.1136/bmj.39489.470347.AD 18436948 PMC2335261

[44] Eakle R Gomez GB Naicker N : HIV pre-exposure prophylaxis and early antiretroviral treatment among female sex workers in South Africa: Results from a prospective observational demonstration project. *PLoS Med.* 2017;14(11):e1002444. 10.1371/journal.pmed.1002444 29161256 PMC5697804

[45] Tabidze I Rusie L Hendry C : Primary and secondary syphilis and pre exposure prophylaxis (PrEP), Chicago, IL, 2014–2016.STD Prevention Conference; Washington, DC,2018.

[46] Giguère K Béhanzin L Guédou FA : PrEP Use Among Female Sex Workers: No Evidence for Risk Compensation. *J Acquir Immune Defic Syndr.* 2019;82(3):257–64. 10.1097/QAI.0000000000002134 31356468 PMC6798737

[47] Maljaars LP Gill K Smith PJ : Condom migration after introduction of pre-exposure prophylaxis among HIV-uninfected adolescents in South Africa: A cohort analysis. *South Afr J HIV Med.* 2017;18(1):712. 10.4102/sajhivmed.v18i1.712 29568635 PMC5843039

[48] Blumenthal J : Results from a PrEP demonstration project for at-risk cisgender women in the US. *Conference on Retroviruses and Opportunistic Infections (CROI)*; Boston, Massachusetts,2020.

[49] Gill K Celum C Breen G : P432 High prevalence and incidence of curable STIs among young women initiating PrEP in a township in south africa.STI & HIV World Congress (Joint Meeting of the 23rd ISSTDR and 20th IUSTI); Vancouver, Canada.2019;95(suppl 1). 10.1136/sextrans-2019-sti.518

[50] Delany-Moretlwe S Mgodi N Bekker LG : O10. 3 High curable STI prevalence and incidence among young african women initiating PrEP in HPTN 082. *STI & HIV World Congress (Joint Meeting of the 23rd ISSTDR and 20th IUSTI)*; Vancouver, Canada,2019;95(1). 10.1136/sextrans-2019-sti.160

[51] Stewart J Omollo V Odoyo J : P424 High prevalence and incidence of bacterial STIs in young women at high risk of HIV prior to PrEP scale-up in kenya. *STI & HIV World Congress (Joint Meeting of the 23rd ISSTDR and 20th IUSTI)*; Vancouver, Canada,2019;95(suppl1). 10.1136/sextrans-2019-sti.510

[52] Van Damme L Corneli A Ahmed K : Preexposure prophylaxis for HIV infection among African women. *N Engl J Med.* 2012;367(5):411–22. 10.1056/NEJMoa1202614 22784040 PMC3687217

[53] Wu H Strong C Ku S : Syphilis acquisition and dosing schedule for pre-exposure prophylaxis (PrEP) users in Taiwan PrEP demonstration project. *22nd International AIDS Conference (AIDS 2018)*; Amsterdam, Netherlands.2018. Reference Source

[54] Clement ME Nicchitta M Sun Y : Preexposure Prophylaxis Outcomes in an Urban Community in North Carolina: Discontinuation of Care and Sexually Transmitted Infections. *Sex Transm Dis.* 2021;48(3):183–8. 10.1097/OLQ.0000000000001288 33003182 PMC7867579

[55] Harrison A Colvin CJ Kuo C : Sustained high HIV incidence in young women in Southern Africa: social, behavioral, and structural factors and emerging intervention approaches. *Curr HIV/AIDS Rep.* 2015;12(2):207–15. 10.1007/s11904-015-0261-0 25855338 PMC4430426

[56] Dellar RC Dlamini S Karim QA : Adolescent girls and young women: key populations for HIV epidemic control. *J Int AIDS Soc.* 2015;18(2 Suppl 1):19408. 10.7448/IAS.18.2.19408 25724504 PMC4344544

[57] Becker ML Bhattacharjee P Blanchard JF : Vulnerabilities at First Sex and Their Association With Lifetime Gender-Based Violence and HIV Prevalence Among Adolescent Girls and Young Women Engaged in Sex Work, Transactional Sex, and Casual Sex in Kenya. *J Acquir Immune Defic Syndr.* 2018;79(3):296–304. 10.1097/QAI.0000000000001826 30113403 PMC6203425

[58] Wamoyi J Stobeanau K Bobrova N : Transactional sex and risk for HIV infection in sub‐Saharan Africa: a systematic review and meta-analysis. *J Int AIDS Soc.* 2016;19(1):20992. 10.7448/IAS.19.1.20992 27809960 PMC5095351

[59] Baeten JM Palanee-Phillips T Mgodi NM : Safety, uptake, and use of a dapivirine vaginal ring for HIV-1 prevention in African women (HOPE): an open-label, extension study. * Lancet HIV.* 2021;8(2):e87–95. 10.1016/S2352-3018(20)30304-0 33539762 PMC8038210

[60] World Health Organization: WHO recommends the dapivirine vaginal ring as a new choice for HIV prevention for women at substantial risk of HIV infection. *WHO News.* 2021. Reference Source

[61] Nel A Van Niekerk N Van Baelen B : Safety, adherence, and HIV-1 seroconversion among women using the dapivirine vaginal ring (DREAM): an open-label, extension study. *Lancet HIV.* 2021;8(2):e77–86. 10.1016/S2352-3018(20)30300-3 33539761

[ref-62] Griffin JB Ridgeway K Montgomery E : Vaginal ring acceptability and related preferences among women in low-and middle-income countries: a systematic review and narrative synthesis. *PLoS One.* 2019;14(11):e0224898. 10.1371/journal.pone.0224898 31703094 PMC6839883

[63] Delany-Moretlwe S Hughes JP Bock P : Long acting injectable cabotegravir is safe and effective in preventing HIV infection in cisgender women: results from HPTN 084. *4th HIV Research for Prevention conference (HIVR4P // Virtual)*;2021. Reference Source

[64] Perez CC : Invisible women: Exposing data bias in a world designed for men.Random House;2019. Reference Source 10.3399/bjgp20X709745 PMC7194768 32354824

[65] Curno MJ Rossi S Hodges-Mameletzis I : A Systematic Review of the Inclusion (or Exclusion) of Women in HIV Research: From Clinical Studies of Antiretrovirals and Vaccines to Cure Strategies. *J Acquir Immune Defic Syndr.* 2016;71(2):181–8. 10.1097/QAI.0000000000000842 26361171

[66] Adimora AA Ramirez C Poteat T : HIV and women in the USA: what we know and where to go from here. *Lancet.* 2021;397(10279):1107–15. 10.1016/S0140-6736(21)00396-2 33617768

[67] World Health Organization: Prevention and control of sexually transmitted infections (STIs) in the era of oral pre-exposure prophylaxis (PrEP) for HIV.Geneva: World Health Organization;2019;1–16. Reference Source

[68] Castro DR Delabre RM Molina JM : Give PrEP a chance: moving on from the "risk compensation" concept. *J Int AIDS Soc.* 2019;22 Suppl 6(Suppl Suppl 6):e25351. 10.1002/jia2.25351 31468693 PMC6715948

[69] Torrone EA Morrison CS Chen PL : Prevalence of sexually transmitted infections and bacterial vaginosis among women in sub-Saharan Africa: An individual participant data meta-analysis of 18 HIV prevention studies. *PLoS Med.* 2018;15(2):e1002511. 10.1371/journal.pmed.1002511 29485986 PMC5828349

[70] Melesse DY Mutua MK Choudhury A : Adolescent sexual and reproductive health in sub-Saharan Africa: who is left behind? *BMJ Glob Health.* 2020;5(1):e002231. 10.1136/bmjgh-2019-002231 32133182 PMC7042602

