# Supplementary material for: Impact of daily, oral pre-exposure prophylaxis on the risk of bacterial sexually transmitted infections among cisgender women: a systematic review and narrative synthesis
Source: Wellcome Open Res. 2022 Aug 15;7:108. Originally published 2022 Mar 23. [Version 2] doi: 10.12688/wellcomeopenres.17457.2 (PMC9391742; doi:10.12688/wellcomeopenres.17457.2)
Supplement: Peer review history

# Peer review: Reviewer response for version 2

**DOI:** 10.21956/wellcomeopenres.20061.r51990

**Published:** 2022-08-19

**Authors:** Janey Sewell

**Affiliations:** 1 UCL Institute for Global Health, University College London, London, UK

All comments have been addressed concisely.

Are the rationale for, and objectives of, the Systematic Review clearly stated?

Yes

Is the statistical analysis and its interpretation appropriate?

Yes

Are sufficient details of the methods and analysis provided to allow replication by others?

Yes

Are the conclusions drawn adequately supported by the results presented in the review?

Yes

Reviewer Expertise:

Infection and population health, HIV.

I confirm that I have read this submission and believe that I have an appropriate level of expertise to confirm that it is of an acceptable scientific standard.

# Peer review: Reviewer response for version 1

**DOI:** 10.21956/wellcomeopenres.19303.r51243

**Published:** 2022-07-12

**Authors:** Katherine Gill

**Affiliations:** 1 Desmond Tutu HIV Centre, University of Cape Town, Cape Town, South Africa

Thank you for the opportunity to review this paper. It's an important topic and a timely review. In general, I thought it was a well-written paper. I only have a couple of minor comments.

**Introduction:**

- Para1: Injectable PrEP is a promising option but seems misplaced here. Focus on oral PrEP and in the discussion you can briefly mention long acting PrEP.
- Para2: Understand what you mean by “syndemic” but don’t think it’s the appropriate term to use when describing HIV prevention. Maybe add that it is a theoretical relationship.
- Para 3: Unprotected sex = condomless sex.

**Methods:**

- Search strategy: No reference made to an african Clinical trial register like the SANCTR or PACTR. This may have biased your results.

**Results:**

- Table 1: Please define the symbols under the study quality column.

**Conclusions: **

- Paragraph 4 - not sure what you mean by 27% of the studies reported risk

Are the rationale for, and objectives of, the Systematic Review clearly stated?

Yes

Is the statistical analysis and its interpretation appropriate?

Yes

Are sufficient details of the methods and analysis provided to allow replication by others?

Yes

Are the conclusions drawn adequately supported by the results presented in the review?

Yes

Reviewer Expertise:

HIV Prevention

I confirm that I have read this submission and believe that I have an appropriate level of expertise to confirm that it is of an acceptable scientific standard.

# Response

**Published:** 2022-07-25

**Authors:** Vasiliki Papageorgiou

**Affiliations:** Imperial College London, UK

We thank both Janey Sewell (Reviewer 1) and Katherine Gill (Reviewer 2) for their supportive comments to help strengthen this article. We have detailed our comments below (point by point) with edits indicated using tracked changes in the manuscript file.

**Comments:**

 Thank you for the opportunity to review this paper. It's an important topic and a timely review. In general, I thought it was a well-written paper. I only have a couple of minor comments.

**Introduction:**

- Para1: Injectable PrEP is a promising option but seems misplaced here. Focus on oral PrEP and in the discussion you can briefly mention long acting PrEP.
- Para2: Understand what you mean by “syndemic” but don’t think it’s the appropriate term to use when describing HIV prevention. Maybe add that it is a theoretical relationship.
- Para 3: Unprotected sex = condomless sex.

**Response/revisions**

- We have split the two sentences here for clarity and have moved the sentence focussing on long-acting PrEP to the Discussion as suggested.

- We use the terminology stated in Gandhi *et al*. (2019) paper here referring to a ‘syndemic’ however agree that this is theoretical so have clarified here. We have also referred to causal relationships here to make this distinction clearer and added a new reference which reflects on the complexity of uncovering the causal pathway between PrEP use and STI risk:
  Stewart, J., Baeten, J.M. HIV pre-exposure prophylaxis and sexually transmitted infections: intersection and opportunity. *Nat Rev Urol* **19, **7–15 (2022). https://doi.org/10.1038/s41585-021-00527-4
- We agree to and have made the suggestion highlighted.  

**Comments:**

**Methods:**

- Search strategy: No reference made to an african Clinical trial register like the SANCTR or PACTR. This may have biased your results.

**Response/revisions**

- We agree that a need remains to highlight this potential bias, so we have included an additional sentence in the Discussion section.

**Comments:**

**Results:**

- Table 1: Please define the symbols under the study quality column.

**Response/revisions**

- We have added a footnote to Table 1 to explain the symbols for the GRADE assessment as well as clarifications for each study rating.

**Comments:**

**Conclusions: **

- Paragraph 4 - not sure what you mean by 27% of the studies reported risk

**Response/revisions**

- We have clarified this sentence in the ‘strengths and limitations’ section of the Discussion to highlight that this refers to changes in sexual behaviour at baseline and follow-up.

# Peer review: Reviewer response for version 1

**DOI:** 10.21956/wellcomeopenres.19303.r50681

**Published:** 2022-06-10

**Authors:** Janey Sewell

**Affiliations:** 1 UCL Institute for Global Health, University College London, London, UK

Thank you for the opportunity to review this systematic review on the "Impact of daily, oral pre-exposure prophylaxis on the risk of bacterial sexually transmitted infections among cisgender women." Overall the manuscript is extremely clear and concise and presents a timely review of the evidence on the impact of PrEP on STI infections among cisgender women. I have just a few queries and clarifications as outlined below.

**Introduction**

 The introduction clearly outlines the context and rationale for this review. Minor comments or clarifications required:

  

1. Paragraph 1:  'PrEP can be administered as a daily, oral tablet or long-acting injection' - I wonder if some explanation to demonstrate the distinctions with vaginal ring trials which would also help contextualise HIV prevention interventions in women, might be useful here or perhaps see point 3).
2. Paragraph 3 'For instance, an intended benefit of PrEP use is the freedom for people to have unprotected sex,' - does the term 'unprotected sex' mean condomless sex? It is important to define 'unprotected sex' in the context of PrEP use (offering protection against HIV) here.
3. Paragraph 6 '...however few trials have reported results specifically on ciswomen, despite accounting for almost half of new HIV infections in adults globally' - could the authors postulate as to why this is? is it (as described in the previous sentence) lack of representation within clinical trials? or fewer trials specifically aimed at women? This could also be where the distinction between PrEP trials and vaginal ring trials might land.
4. Paragraph 7 - acronyms for PrEP and STIs have already been defined in paragraph 1, I am not sure about the added value for defining again here. However, as in the first paragraph of the introduction, there has been a description of the long-acting injectable, cabotegravir, it would be worth clarifying that this review focuses on oral PrEP rather than injectable PrEP.

**Methods**

 The methods are transparent and comprehensible. It would be useful to expand on (in the 'Risk of Bias' section where GRADE is referred to) the four levels of evidence that are produced by a GRADE assessment and how these relate to the symbols in Table 1.

**Results**

 The results are clearly presented. Some clarifications are needed.

1. It would be useful in the 'Study characteristics' section for the studies to be referenced as they have been throughout the rest of the results section. 
2. Table 1 - it would be useful to know how the reported outcomes were collected - lab/clinician confirmed/diagnoses? self-reported?
3. Table 1 - GRADE symbols need to have been described in the Methods section so that this column is interpretable to anyone not familiar with GRADE.  
4. Table 2 - column % change - please add (in the footnote or text) how this calculation was made.
5. Synthesis of results: sexual behaviour - are all these behaviours self-reported (assume so!)? - this should be made clear in this section.
6. Quality assessment. This section needs to be contextualised by outlining the scoring for GRADE as per the comment in the Methods section. A summary of how many studies scored ++, and how many scored +, would contextualise the first sentence 'Overall, the quality of evidence was low' - and (as 9/11 studies were assessed as ‘+’ is it not more accurate to say that 'Overall, the quality of evidence was very low'?

**Discussion**

 The discussion is succinct and describes the results clearly in light of the limited evidence. A few minor details to consider:

1. Strengths and limitations: 'Secondly, several of the included studies were very small and some of poor quality' - Table 1 would indicate that they were all of 'low' or 'very low' quality. 
2. Paragraph 4: 'Just over one-quarter of the included studies reported risk at follow-up and baseline' - it is not clear what 'risk' refers to here. 

Otherwise no further comments or recommendations.

Are the rationale for, and objectives of, the Systematic Review clearly stated?

Yes

Is the statistical analysis and its interpretation appropriate?

Yes

Are sufficient details of the methods and analysis provided to allow replication by others?

Yes

Are the conclusions drawn adequately supported by the results presented in the review?

Yes

Reviewer Expertise:

Infection and population health, HIV.

I confirm that I have read this submission and believe that I have an appropriate level of expertise to confirm that it is of an acceptable scientific standard, however I have significant reservations, as outlined above.

# Response

**Published:** 2022-07-25

**Authors:** Vasiliki Papageorgiou

**Affiliations:** Imperial College London, UK

We thank both Janey Sewell (Reviewer 1) and Katherine Gill (Reviewer 2) for their supportive comments to help strengthen this article. We have detailed our comments below (point by point) with edits indicated using tracked changes in the manuscript file.

**Comments:**

 Thank you for the opportunity to review this systematic review on the "Impact of daily, oral pre-exposure prophylaxis on the risk of bacterial sexually transmitted infections among cisgender women." Overall the manuscript is extremely clear and concise and presents a timely review of the evidence on the impact of PrEP on STI infections among cisgender women. I have just a few queries and clarifications as outlined below.

**Introduction**

 The introduction clearly outlines the context and rationale for this review. Minor comments or clarifications required:

1.  Paragraph 1:  'PrEP can be administered as a daily, oral tablet or long-acting injection' - I wonder if some explanation to demonstrate the distinctions with vaginal ring trials which would also help contextualise HIV prevention interventions in women, might be useful here or perhaps see point 3).
2. Paragraph 3 'For instance, an intended benefit of PrEP use is the freedom for people to have unprotected sex,' - does the term 'unprotected sex' mean condomless sex? It is important to define 'unprotected sex' in the context of PrEP use (offering protection against HIV) here.
3. Paragraph 6 '...however few trials have reported results specifically on ciswomen, despite accounting for almost half of new HIV infections in adults globally' - could the authors postulate as to why this is? is it (as described in the previous sentence) lack of representation within clinical trials? or fewer trials specifically aimed at women? This could also be where the distinction between PrEP trials and vaginal ring trials might land.
4. Paragraph 7 - acronyms for PrEP and STIs have already been defined in paragraph 1, I am not sure about the added value for defining again here. However, as in the first paragraph of the introduction, there has been a description of the long-acting injectable, cabotegravir, it would be worth clarifying that this review focuses on oral PrEP rather than injectable PrEP.

**Response/revisions**

1. To highlight that our study focusses on oral PrEP only, we have moved detail on other modalities into the Discussion and have clarified that we are unable to draw conclusions on these methods (as a limitation). We have included more information on vaginal ring trials and other modalities of PrEP in the Discussion, including 4 additional references:
  Baeten JM, Palanee-Phillips T, Mgodi NM, Mayo AJ, Szydlo DW, Ramjee G, Mirembe BG, Mhlanga F, Hunidzarira P, Mansoor LE, Siva S. Safety, uptake, and use of a dapivirine vaginal ring for HIV-1 prevention in African women (HOPE): an open-label, extension study. The Lancet HIV. 2021 Feb 1;8(2):e87-95.
  World Health Organization. WHO recommends the dapivirine vaginal ring as a new choice for HIV prevention for women at substantial risk of HIV infection. WHO News. January. 2021.
  Nel A, Van Niekerk N, Van Baelen B, Malherbe M, Mans W, Carter A, Steytler J, Van der Ryst E, Craig C, Louw C, Gwetu T. Safety, adherence, and HIV-1 seroconversion among women using the dapivirine vaginal ring (DREAM): an open-label, extension study. The Lancet HIV. 2021 Feb 1;8(2):e77-86.
  Griffin JB, Ridgeway K, Montgomery E, Torjesen K, Clark R, Peterson J, Baggaley R, van der Straten A. Vaginal ring acceptability and related preferences among women in low-and middle-income countries: a systematic review and narrative synthesis. PloS one. 2019 Nov 8;14(11):e0224898.
2.  As also suggested by reviewer 2, we have decided to clarify the wording here as condomless sex rather than ‘unprotected sex’.
3. We have included additional information here about the exclusion of pregnant, breastfeeding and women of childbearing potential from PrEP trials – and reference:
  Davey DL, Bekker LG, Bukusi EA, Chi BH, Delany-Moretlwe S, Goga A, Lyerly AD, Mgodi NM, Mugo N, Myer L, Noguchi LM. Where are the pregnant and breastfeeding women in new pre-exposure prophylaxis trials? The imperative to overcome the evidence gap. The Lancet HIV. 2022 Jan 25.
4. We have removed the acronyms as suggested and made it clearer that this review focuses on oral PrEP.

**Comments:**

**Methods**

 The methods are transparent and comprehensible. It would be useful to expand on (in the 'Risk of Bias' section where GRADE is referred to) the four levels of evidence that are produced by a GRADE assessment and how these relate to the symbols in Table 1.

**Response/revisions**

 We agree that additional information on how the GRADE tool is used would be helpful for readers so have included additional information about how to interpret ratings/assessment, including a new reference:

 Schünemann HJ, Higgins JP, Vist GE, Glasziou P, Akl EA, Skoetz N, et al. Chapter 14: Completing ‘Summary of findings’ tables and grading the certainty of the evidence. In: *Cochrane Handbook for Systematic Reviews of Interventions*. Version 6.3. 2022. https://training.cochrane.org/handbook/current/chapter-14

**Comments:**

**Results**

 The results are clearly presented. Some clarifications are needed.

1. It would be useful in the 'Study characteristics' section for the studies to be referenced as they have been throughout the rest of the results section. 
2. Table 1 - it would be useful to know how the reported outcomes were collected - lab/clinician confirmed/diagnoses? self-reported?
3. Table 1 - GRADE symbols need to have been described in the Methods section so that this column is interpretable to anyone not familiar with GRADE.  
4. Table 2 - column % change - please add (in the footnote or text) how this calculation was made.
5. Synthesis of results: sexual behaviour - are all these behaviours self-reported (assume so!)? - this should be made clear in this section.
6. Quality assessment. This section needs to be contextualised by outlining the scoring for GRADE as per the comment in the Methods section. A summary of how many studies scored ++, and how many scored +, would contextualise the first sentence 'Overall, the quality of evidence was low' - and (as 9/11 studies were assessed as ‘+’ is it not more accurate to say that 'Overall, the quality of evidence was very low'?

**Response/revisions**

 1. We have added references to clarify which studies we are referring to here.

 2. We have added how reported outcomes were collected for each study.

 3. Agreed. We have added a footnote to Table 1 to explain the symbols for the GRADE assessment as well as clarifications for each study rating.

 4. Percentage change calculation added as a footnote.

 5. We have added a sentence in the opening paragraph of the “synthesis of results: sexual behaviour” section to clarify how this was measured across studies. We have also added detail in Table 1 under outcome column.

 6. We have added a sentence outlining the number of studies which scored very low and low quality with the GRADE assessment in this sub-section.

**Comments:**

**Discussion**

 The discussion is succinct and describes the results clearly in light of the limited evidence. A few minor details to consider:

1. Strengths and limitations: 'Secondly, several of the included studies were very small and some of poor quality' - Table 1 would indicate that they were all of 'low' or 'very low' quality. 
  Paragraph 4: 'Just over one-quarter of the included studies reported risk at follow-up and baseline' - it is not clear what 'risk' refers to here. 

Otherwise no further comments or recommendations.

**Response/revisions**

1. We have amended this sentence to match the wording of the GRADE assessment.
2. We have clarified this sentence in the ‘strengths and limitations' section of the Discussion to highlight that this refers to changes in sexual behaviour at baseline and follow-up.
